# Challenges in non-communicable disease mitigation among community health workers: A scoping review

**DOI:** 10.4102/jphia.v16i1.1494

**Published:** 2025-10-31

**Authors:** Nongiwe L. Mhlanga, Sikhumbuzo A. Mabunda

**Affiliations:** 1 Department of Public Health, Faculty of Medicine and Health Sciences, Walter Sisulu University, Mthatha, South Africa; 2 School of Public Health, University of New South Wales, Sydney, Australia; 3 The George Institute of Public Health, University of New South Wales, Sydney, Australia

**Keywords:** task-sharing, community health workers, non-communicable diseases, Africa, challenges, health workforce

## Abstract

**Background:**

There is an increase in non-communicable diseases (NCDs) in Africa, amid a high health worker shortage, necessitating task-sharing with community health workers (CHWs). However, task sharing with CHWs may not have positive patient outcomes, as they face several challenges.

**Aim:**

To describe the task-sharing challenges faced by CHWs in NCDs mitigation.

**Setting:**

Studies conducted in Africa were selected.

**Method:**

The Arksey and O’ Marley Framework was used. Included articles were published in English from 2015 to 2025. PubMed, ScienceDirect, and Google Scholar were searched from 26 March 2025. Two reviewers used Covidence to select studies, and conflicts were resolved through discussions. The researchers developed the data extraction tool and used content analysis to analyse data.

**Results:**

Articles screened by title were 189, with a final selection of 14 articles. The review found that an individual-level challenge was a lack of skills and inadequate knowledge. Organization-level challenges included a lack of supervision, a lack of equipment and infrastructure, and a poor referral system. Community-level challenges included safety concerns, poverty among community members, lack of transport, and mistrust of community health workers.

**Conclusion:**

It is essential to capacitate CHWs through continued supervision and training, and with policies that address broader socio-economic challenges like poverty and crime in Africa.

**Contribution:**

The study contributes to increasing the efficiency of the African CHWs by providing insights into the challenges they experience so that these challenges may be addressed.

## Introduction

The non-communicable disease (NCD) burden poses a significant public health challenge, accounting for more than 82% of all premature deaths in low- and middle-income countries (LMICs).^1^ The highest risk of premature deaths from NCDs is in sub-Saharan Africa.^2^ Worsening this problem is a shortage of health workers to manage and prevent NCDs. By 2022, it was estimated that health workers would cover only 43% of Africa’s population’s health service needs.^3^ To curb the burden, the World Health Organization describes the use of task-sharing to increase the capacity and efficiency of the health workforce.^4^ Although task-sharing offers a solution to manage NCDs, there is evidence highlighting challenges such as the complexity of tasks faced by community health workers (CHWs), which may affect patient outcomes in NCD mitigation. Therefore, this review sought to describe the task-sharing challenges faced by CHWs in NCD mitigation in Africa.

In Africa, CHWs form an integral part of health service delivery and have aided other initiatives such as human immunodeficiency virus (HIV) care.^5,6,7^ Community health workers are non-professional health workers who have received less formal education and training than professional health workers and work voluntarily or for a stipend while residing in the communities they serve.^8,9^ Community health workers may also be referred to as lay health workers.^10,11,12^ or village health workers,^13^ and in this study are referred to as CHWs. The persistent challenge of health worker shortages, with consequent pertinence to implement task-sharing or task-shifting, has resulted in the growing recognition of the work performed by CHWs.^14^ Task-sharing refers to the distribution of tasks to health workers with lesser duration of training and qualifications to maximise the health workforce, with these delegated tasks being performed collaboratively.^15^

Previous systematic reviews have shown that tasks shared with CHWs include the management and screening for hypertension,^16^ diabetes health education, screening, support and advocacy.^9,17^ Regarding mental health service provision, CHWs in Malawi have also delivered psychosocial interventions for severe and common mental health conditions.^18^ In some cases, interventions for managing NCDs have resulted in better patient outcomes than the usual care provided by professional health workers. For example, in Zimbabwe, a psychosocial intervention delivered by CHWs resulted in improved symptoms compared with usual care.^19^ On the other hand, a South African randomised controlled trial reported that task-sharing in providing mental healthcare for antenatal depression was not effective.^20^ Given the poor patient outcomes reported in the South African study, while also recognising that positive patient outcomes do not mean that there are no challenges experienced, it is imperative to describe the challenges experienced in NCD mitigation service provision.

In some LMICs, comparable to Africa, some challenges include health policy-related issues, such as a lack of standardised training and government support,^21,22^ or institutional factors such as inadequate guidance reported in an Indonesian study.^23^ Moreover, there could be personal challenges experienced by the CHWs, such as resistance from family and a lack of competency because of inadequate training and knowledge.^24,25^ Despite this, CHWs still contribute to the overall management of NCDs, and it is important to understand and address these challenges to improve patient outcomes and facilitate the capacitation of health workers. Describing these challenges also enables the development of policies and improves the functioning of health systems for mitigating the adverse outcomes of NCDs.

Past scoping reviews have outlined the barriers and enablers faced by CHWs in managing mental health conditions in all countries,^24^ and there is also evidence from studies conducted outside Africa.^25^ However, there remains a gap in the evidence synthesis from Africa, which is faced with an increasing burden of NCDs and a shortage of health workers. Therefore, the scoping review question was: What are the task-sharing challenges faced by CHWs in NCD mitigation in Africa? Secondary research questions were: in what contexts do CHWs face task-sharing challenges in NCD mitigation in Africa, and what are the characteristics of CHWs facing task-sharing challenges in NCD mitigation? Before conducting this scoping review, a preliminary search of the databases, ScienceDirect and PubMed was conducted, and no similar topic was found. A scoping review, rather than a systematic review, was selected because the authors sought to clarify a concept, ‘challenges experienced by CHWs in Africa in NCD mitigation’, and not provide evidence for a clinical question.^26^ The protocol for this scoping review was registered on the Open Science Framework, registration number https://osf.io/rmgns/.

## Methods

The Arksey and O’Malley Framework for conducting scoping reviews was used.^27,28^ The study was reported according to the Preferred Reporting Items for Systematic reviews and Meta-Analyses extension for Scoping Reviews (PRISMA-ScR).^29^

### Eligibility criteria

The review’s eligibility criteria were developed using the Population, Concept and Context (PCC) framework.

Inclusion criteria:

- Population: The population of interest in this study was CHWs who shared tasks in mitigating NCDs.
- Context: The included studies described African settings or communities (urban, rural, peri-urban) where CHWs provided NCD mitigation services.
- Concept: The review included studies that described the challenges in task-sharing faced by CHWs in the mitigation of NCDs.

Types of articles included:

- Articles published between 2015 and 2025 to maintain the significance of the findings.
- Articles published in English to minimise translation bias.
- Peer-reviewed studies that used any study design, such as systematic reviews, qualitative studies, quantitative studies and mixed methods designs.

Types of articles excluded:

- Institutional reports and grey literature were excluded because of difficulties in assessing the quality of the reports and as some of the grey literature may not have been based on empirical studies.
- Systematic reviews that included studies outside Africa.
- Articles not published in English to minimise translation bias.

### Literature search

A three-step process was used to select studies.^27,28^ Three databases, such as PubMed, ScienceDirect and Google Scholar (first 10 pages), were searched. These databases were selected as they have been previously used in other African systematic reviews.^30,31^ The search was conducted using the key terms ‘task-sharing’, ‘community health workers’, ‘challenges’, ‘non-communicable diseases’ and ‘Africa’. After the relevant articles were identified, the index terms were used to develop a search strategy for the databases. The database search was conducted on 26 March 2025. The Boolean operators used were ‘OR’ and ‘AND’. The full search strategies for all three databases are included in Online Appendix 1. The reference lists of the selected articles were also searched for relevant studies.

### Selection of studies

The references of the identified studies were uploaded to the Mendeley reference manager, and duplicates were removed. Titles and abstracts were then screened by two reviewers, N.L.M. and N.F. Screening was performed using Covidence, whereby potential studies and their full texts were screened against the eligibility criteria. During screening, reasons for exclusion were noted. Disagreements between the reviewers were resolved through discussion. The PRISMA chart was used to show the decision process.^32^

### Data extraction

Data were extracted from selected articles by two reviewers using a data extraction tool developed by the reviewers. The data extracted included the title of the study, year of publication, country where the study was conducted, objectives of the study, study design, sample size and outcomes in relation to the review. The data extraction tool is included as Online Appendix 2.

### Quality appraisal of the selected studies

The selected studies were appraised using the Critical Appraisal Skills Programme (CASP) checklists. Although the Arksey and O’Malley Framework does not require the appraisal of selected studies for inclusion, the researchers deemed it necessary to appraise the studies. Two checklists were used, and these were for qualitative studies^33^ and cross-sectional studies.^34^ The reviewers, N.L.M. and N.F., appraised the studies independently, and any disagreements were discussed. The CASP checklists broadly assessed the validity of the results, results and the usefulness of the results. The overall decision would be negative (poor methodology) or positive (sound methodology).

### Data analysis

A numeric analysis was first conducted of the selected studies. To answer the research questions, qualitative content analysis was used to analyse data independently by two researchers. Four steps outlined by Kleinheksel et al.^35^ were used. The first step was identifying the unit of meaning aligned with the main study objective. The second step was labelling similar units with a code. The third step was grouping the codes into a category, and lastly (the fourth step), the categories were organised as themes.

## Review findings

### Search results

**FIGURE 1:**
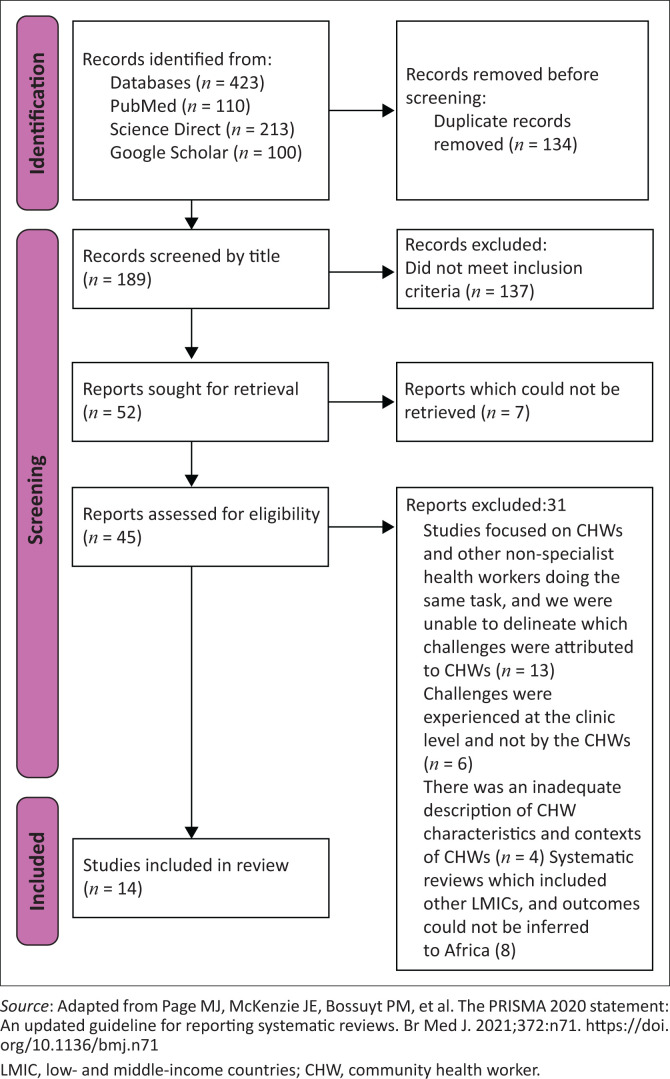
The Preferred Reporting Items for Systematic reviews and Meta-Analyses flow chart.

A total of 189 articles were screened by title only, of which 137 did not meet the inclusion criteria, and 7 could not be retrieved. As such, 45 articles were screened by title and abstract. After screening, 14 articles were selected. Figure 1 shows the PRISMA flow chart of the decision process.

### Results from the quality appraisal

All the 11 studies assessed using the CASP qualitative checklist had an overall appraisal summary of positive methodological quality. The most common unreported item in the qualitative studies was the relationship between participants and researchers. The two studies that used a mixed methods design were assessed using the CASP cross-sectional and qualitative checklists. The overall appraisal decision for both studies was ‘positive’. One study was assessed using the CASP checklist for cross-sectional studies, and the overall decision was also positive. The outcomes of the quality appraisal are included as Online Appendix 3.

### Characteristics of the selected studies

Fourteen (*n* = 14) studies were included in the review. Most (28.6%, *n* = 4) studies were from South Africa, three (21.4%) studies from Uganda, two (14.3%) studies from Nigeria and one (7.1%) study each from Kenya, Zimbabwe, Mozambique and Ethiopia. One (7.1%) study reported findings from South Africa, Mexico, Bangladesh and Guatemala. Selected studies were published between 2015 and 2024, with most (four each) being published in 2023 and 2022. Eleven (78.6%) studies used a qualitative approach, while two studies used mixed methods and one used a quantitative approach. Nine (64.3%) studies focused on mental health, while other studies reported on hypertension (7.1%), diabetes and hypertension (7.1%), cardiovascular diseases (7.1%), all NCDs (7.1%) and chronic obstructive pulmonary disease (COPD) (7.1%). Table 1 summarises the characteristics of the selected studies.

**TABLE 1:** Summary of selected studies.

Study	Objective	Country	Study design	Sample size	Challenges experienced by the CHWs
Abrahams-Gessel et al.^36^	To assess CHW performance during training and explore the fieldwork and training experience.	South Africa, Bangladesh, Guatemala and Mexico	Qualitative study	64	Safety concerns: crime.
Ajisegiri et al.^37^	To explore the practices and roles of CHWs in providing hypertension and diabetes care.	Nigeria	Mixed methods	77	Referral challenges. Inadequate space and equipment. Lack of supervision.
Sensoy Bahar et al.^38^	To examine the experiences of CHWs and parent peers with a family strengthening intervention.	Uganda	Qualitative study	95	Lack of skills and knowledge.
Cumbe et al.^39^	To examine the knowledge, attitudes and practices of CHWs towards epilepsy.	Mozambique	Quantitative study	135	Lack of skills and knowledge.
Davies et al.^40^	To assess the process in the delivery of counselling and make recommendations for task-sharing.	South Africa	Qualitative study	39	Lack of skills and knowledge. Poverty among community members.
Doresha and Mash^41^	To explore the role of CHWs for NCDs in the Cape Town Eastern District.	South Africa	Qualitative study	9	Challenges with the referral. Lack of knowledge and skills.
Ingenhoff et al.^42^	To describe the barriers and facilitators in CHW-led chronic obstructive pulmonary disease screening and referral.	Uganda	Qualitative study	52	Challenges with the referral. Inadequate equipment and infrastructure. Community mistrust.
Karungi et al.^43^	To assess the 5-day training of CHWs in Uganda in the care and management of people with Dementia.	Uganda	Qualitative study	30	Lack of knowledge and skills. Challenges with referral.
Kidia et al.^44^	To understand the sustainability, feasibility and acceptability of a mental health intervention implemented by CHWs.	Zimbabwe	Qualitative study	32	Poverty among community members. Transport challenges.
Munodawafa et al.^45^	To explore perceptions of counsellors who delivered a task-sharing psychosocial counselling intervention.	South Africa	Qualitative study	6	Lack of knowledge and skills. Safety concerns -crime. Poverty among community members.
Ojagbemi et al.^46^	To assess the experiences of CHWs providing care to older people with depression.	Nigeria	Qualitative study	24	Transport challenges.
Selohilwe et al.^47^	To describe the multilevel factors affecting the implementation and potential dissemination of a service for depression.	South Africa	Qualitative study	86	Lack of knowledge and skills. Inadequate infrastructure and equipment. Inadequate supervision.
Teshome et al.^48^	To explore the enablers and barriers affecting CHW hypertension screening.	Ethiopia	Qualitative study	26	Inadequate skills and knowledge. Lack of equipment and infrastructure. Lack of supervision.
Wall et al.^49^	To explore the experiences of CHWs by focusing on their identity, motivation, self-efficacy, stress and burnout.	Kenya	Qualitative study	20	Lack of trust in CHWs. Poverty among community members.

*Note:* Please see the full reference list of the article Mhlanga NL, Mabunda SA. Challenges in non-communicable disease mitigation among community health workers: A scoping review. J Public Health Africa. 2025;16(1), a1494. https://doi.org/10.4102/jphia.v16i1.1494, for more information.

CHW, community health worker; NCD, non-communicable disease.

### Contexts in which community health workers faced challenges in non-communicable disease mitigation service provision

The studies reviewed described the contexts in which CHWs experienced challenges. In most studies, CHWs experienced challenges while working in rural areas.^39,42,44,48^ In some studies, the challenges were experienced in peri-urban communities.^36,40^ One study was conducted in both rural and urban settings.^46^ Additionally, some studies named the study sites: one study^37^ was conducted in four states in Northern and Southern Nigeria, and another study^38^ was conducted in South-West Ugandan schools, while another study was conducted in Mbarara District, Uganda.^43^ Munodawafa et al.^45^ and Selohilwe et al.^47^ conducted their studies in South Africa; Khayelitsha, Cape Town and Kenneth Kaunda District, respectively. One study described the study setting as a low socio-economic area.^41^

### Characteristics of community health workers facing task-sharing challenges in mitigating non-communicable diseases

The studies reviewed described the socio-demographic characteristics of CHWs who experienced challenges in task-sharing for NCD mitigation. In most studies,^36,37,38,41,42,43,46,48^ the CHWs who participated were female. In one Mozambican study,^39^ more male CHWs participated, while in another study,^49^ there was an equal proportion of male and female participants.

Concerning the level of education of the CHWs, four studies^39,40,43,45^ reported that CHWs had completed high school education. In some studies, CHWs had some high school education, such as Abrahams-Gessel et al.^36^ who noted that the minimum level of education was Grade 8, while Wall et al.^49^ note that 45.0% of the CHWs had some high school education. In one rural Ugandan study,^42^ most (70.0%) CHWs had primary school education, while on the other hand, most CHWs in a Nigerian study^37^ had completed a basic national diploma.

### Challenges experienced by community health workers

The review found three themes of challenges experienced by CHWs. These were individual-level, organisation-related and community-related challenges.

### Individual-level challenges

At an individual level, the CHWs’ lack of knowledge and skills affected their ability to mitigate NCDs.

### Inadequate knowledge and skills on non-communicable diseases

Studies^38,39,40,41,43,45,47,48^ included described inadequate knowledge, which made it difficult for CHWs to provide health education, and this lack of knowledge would culminate in poor skills in providing NCD care. In South Africa, Doresha and Mash broadly described the issue of a lack of skills in supporting the management of all NCDs.^41^ For example, the CHWs explained their inability to educate community members on insulin self-administration for diabetes care as they had not been trained.^41^ Other South African studies noted that despite training and having a manual, the CHWs often misinterpreted the therapeutic terms or modalities,^40^ and it was challenging to provide psychosocial care, as mental health was associated with multimorbidity.^47^ Likewise, Ethiopian participants explained that they had inadequate knowledge to answer hypertension-related questions from the community and lacked the skills to measure blood pressure.^48^ Mozambican CHWs also had inadequate knowledge in three areas: (1) believed in false treatments of epilepsy, (2) had inadequate knowledge of its causes, and (3) believed in some cultural treatments.^39^ Although CHWs did not have adequate knowledge on the three issues, Cumbe et al. note that, generally, CHWs had sufficient knowledge of epilepsy.^39^ In Uganda, even after dementia training, CHWs shared how they were still not proficient.^43^ Sometimes, the issue of poor skills was attributed to anxiety about performing a new task and would eventually resolve, with performance improving with continued service provision; however, in a few cases, CHWs’ performance may remain poor.^45^ Poor skill levels at the onset of providing a counselling intervention would only be resolved in the third or fifth session.^38^

### Organisation-level challenges

Organisation-level challenges described in the reviewed articles emanated from the health facilities that supported the CHWs. These organisational challenges included referral problems, inadequate infrastructure and a lack of supervision.

### Referral problems to and from health facilities

Two Ugandan studies,^42,43^ a South African study^41^ and a Nigerian study,^37^ described problems with the referral process. In South Africa, some primary healthcare facilities disregarded referrals made by CHWs who had screened patients for hypertension and diabetes.^41^ Sometimes, community members in South Africa would be referred to health centres, and they would not afford the transport costs.^41^ Similarly, in rural Uganda, CHWs noted how community members screened for COPD would not reach the health centres.^42^ One Ugandan CHW noted, ‘We expect the patients to reach the … clinic and they don’t’.^42^ Moreover, in Uganda and South Africa, referrals were problematic as the service required was unavailable at the clinics.^41,43^ In Nigeria, Ajisegri et al. also noted how CHWs would refer community members to district hospitals; however, there was no feedback about the referred patients, which compelled them to call community members for an update.^37^

### Inadequate infrastructure and equipment in the health facilities

Another organisational challenge experienced by CHWs was the lack of working space and inadequate equipment.^37,42,47,48^ In a South African study, it was revealed that CHWs would improvise for working space, and consequently, patients would not know where to find them for follow-up mental health services.^47^ The lack of space was underscored when CHWs screened for COPD in the homes of community members in rural Uganda and found it difficult to maintain privacy with neighbours who were curious about the spirometry machine.^42^

Regarding the lack of equipment, a community-based hypertension screening intervention in Ethiopia lacked sphygmomanometers and stethoscopes from the health posts, which affected service delivery.^48^ Additionally, CHWs had to ‘improvise’ to measure blood pressure or blood glucose by sharing the sphygmomanometers between departments and asking patients to pay a fee for blood glucose testing.^37^

### Lack of supervision

Three studies^37,47,48^ described the lack of supervision of CHWs. Teshome et al.^48^ found that five participants explained that supervision is essential for programme monitoring. Similarly, in South Africa, irregular and inadequate supervision were identified as a barrier to scaling up mental health service delivery.^47^ One South African CHW explained the issue of irregular supervision: ‘… they take a while to come back again’.^47^ These findings are also quantified in the Nigerian study, which found that 52.2% of CHWs reported that inadequate supervision was a challenge.^37^

### Community-level challenges

Four community-level challenges were identified, namely a lack of trust in CHWs, concerns of neighbourhood crime, transport challenges and poverty among community members.

### Mistrust of community health workers

Although CHWs broadly operate from the communities they come from, in some studies,^42,49^ there were challenges with community members trusting them. In rural Uganda, the CHWs reported that they were not trusted by the community, as they thought they were land grabbers.^42^ The Kenyan study reiterates this issue, highlighting how the community may perceive that their problems would not be kept in confidence when counselling is provided by CHWs.^49^ This lack of trust in CHWs also made it difficult to include family members who were deemed essential for the counselling intervention.^49^

### Concerns of neighbourhood crime (safety)

In South Africa, two studies by Munodawafa et al.^45^ and Abrahams-Gessel^36^ described the issue of crime in the neighbourhoods from which CHWs operated. One South African CHW highlighted, ‘We saw skollies [*thugs*] when we were at the station and had to turn back’.^45^ Similarly, in Cape Town, CHWs who often wear uniforms stood out and were often targets of theft.^36^

### Transport challenges in ensuring community access to services

In a rural Zimbabwean study, one of the barriers to ensuring community access to mental health services through the Friendship Bench initiative was the issue of transport.^44^ Kidia et al. explain that CHWs would often travel long distances in extreme weather and at times on foot.^44^ Similarly, in Kenya, CHWs also provided mental health services to elderly people and shared how they were unable to conduct home visits because of transport challenges.^46^

### Poverty among community members

Community health workers who provided mental health interventions were affected by poverty described by community members.^40,44,45,49^ In the rural Zimbabwean study, some patients had to borrow money to access CHWs for mental health support.^44^ Similarly, South African CHWs felt helpless when faced with patients with material needs, and they were unable to offer such material assistance.^40,45^ Sometimes, there would be progress in mood improvement; however, this would be disrupted by an urgent financial challenge.^40^ The Kenyan study further described how it was difficult to provide a mental health intervention when community members experience financial challenges and cannot afford food.^49^

## Implications and recommendations

This study sought to describe the task-sharing challenges faced by CHWs in Africa in NCD mitigation. The other objectives were to describe the contexts in which CHWs experienced these challenges and the characteristics of CHWs experiencing these challenges. The review found that most studies were conducted in rural contexts, focused on mental health conditions and reported that CHWs were predominantly female and had secondary school education. Task-sharing challenges experienced included inadequate knowledge and lack of skills, referral issues, inadequate infrastructure and equipment and lack of supervision. Community-level challenges were mistrust of CHWs, safety concerns from crime, transport challenges and poverty among community members.

The finding that inadequate knowledge and a lack of skills affected CHWs’ mitigation of NCDs parallels another scoping review^24^ conducted with studies selected globally, as well as another study conducted in Indonesia.^25^ The review by Thobane et al.^24^ also noted that most studies included described a lack of skill. Similarly, the Indonesian study^25^ notes that inadequate skills and knowledge are characterised by an inability to comprehend the terminology used in NCD management, lack of competency and misconceptions about some NCDs. To mitigate the lack of knowledge and skills in NCD management, continued support and training for CHWs should be provided. Innovative approaches across other LMICs are in the form of digital health technologies such as smartphone applications, which can be leveraged to assist CHWs.^50^ Digital health technologies, as indicated in the systematic review by Mishra et al.,^51^ may be used for training and as decision support systems in the management of mental health conditions.

The study also found that there are challenges with referrals made by CHWs. These referral challenges may depict the poor support systems for CHWs in the primary healthcare facilities described in a Nepalese study.^52^ Poor support from primary healthcare facilities also manifested through inadequate supervision. This finding also aligns with an Indonesian study, which noted how CHWs found poor supervision as a challenge in NCD management.^23^ This inadequate supervision may also reflect the broader issue of the health worker shortage^3^ with consequent inadequate capacity to supervise CHWs, often manifesting as competing responsibilities in providing clinical care and supervision.^53^ From this, it is recommended that primary healthcare systems ought to develop support systems for CHWs that also ensure adequate supervision, with consideration for the high workload faced by CHW supervisors. In this regard, digital health technologies can also be leveraged to support the supervision of CHWs and ensure quality NCD management.^51^

Some challenges highlighted in the review, such as a lack of equipment, a lack of transport and poverty in communities, also reflect on the broader challenges faced by some African healthcare workers, especially in marginalised rural contexts.^54^ For example, a study conducted in Zimbabwe notes that rural health facilities may lack ambulances and equipment and that patients may travel more than 5 km to access health services. This is also reflected in this study, where other socio-economic challenges impacted health service delivery. The impact of the socio-economic environment on African health systems may also explain their failures to meet the sustainable development goals (SDGs), in this case, SDG 3 of attaining good health and well-being by reducing premature mortality from NCDs.^55^ Notably, the lack of equipment and poor working conditions characteristic of many African health systems may also be a push factor in the brain drain of health workers,^56^ which worsens the health worker shortages, with consequent inadequate supervision of CHWs. To resolve this, African governments ought to address macroeconomic issues and support communities to deliver healthcare services. The support may include partnerships with not-for-profit organisations that are known to support CHW initiatives through training, financing and providing technical expertise.^57^ In addition, the study found that CHWs were affected by crime in the community. However, the issue was only described in studies conducted in South Africa. A previous study also notes the high levels of crime in South Africa,^58^ which could be a context-specific challenge. Given this, the findings may not truly depict the broader African context, limiting their applicability. Nevertheless, this may also reflect a research gap in other contexts, and we recommend further exploration of this challenge in countries outside South Africa to confirm whether this finding can be transferred to other African contexts.

## Conclusion

The increase in NCDs in Africa, which requires an increase in the capacity of the health workforce, may be affected by poor patient outcomes when tasks are assigned to less qualified workers, such as CHWs. This review sought to describe the African task-sharing challenges experienced by CHWs in NCD mitigation. These challenges included individual-level issues, organisational challenges and problems in the community, such as poverty, lack of transport, crime and mistrust of CHWs. From this review, it is recommended that African governments consider supporting CHW programmes by addressing broader societal challenges such as transport, crime and poverty to support health service delivery. Moreover, primary healthcare facilities should have support systems that leverage digital health technologies for adequate and regular supervision and training of CHWs. Further research could explore safety concerns in the community when CHWs provide services in other African contexts outside South Africa. This study was limited by a lack of thematic saturation on this issue of safety concerns, as only South African studies highlighted the issue. In addition, the study was limited by the inclusion of most (64.3%) studies, which focused on mental health conditions, and this may affect the applicability of findings to other NCDs such as diabetes, hypertension, COPD and cancers. Therefore, it is recommended that future studies should explore the challenges CHWs experience in mitigating other NCDs. The study also excluded grey literature such as institutional reports, dissertations and conference proceedings, which limited the articles included in the study. Notwithstanding these limitations, the study’s main strength was the diversity of the African contexts represented, which enabled evidence synthesis.
